# The Natural History and Outcomes of the Patients with Carcinosarcoma Involving Kidney and Renal Pelvis

**DOI:** 10.1155/2011/693964

**Published:** 2011-07-20

**Authors:** Jue Wang, Fen Wei Wang, Anne Kessinger

**Affiliations:** ^1^Division of Oncology/Hematology, Department of Internal Medicine, University of Nebraska Medical Center, Omaha, NE 68198-7680, USA; ^2^Department of Internal Medicine, Creighton University Medical School, Omaha, NE 68131, USA

## Abstract

*Background*. The objective of this paper was to examine the epidemiology, natural history, and prognostic factors of carcinosarcoma of the kidney and renal pelvis (CSKP) using population-based registry. *Patients and Methods*. Forty-three patients with CSKP, diagnosed between January 1973 and December 2007, were identified from the national Surveillance, Epidemiology, and End Results (SEER) database and reviewed. *Results*. 79% of all patients with known SEER stage were classified as having regional or distant stage; almost all the patients with known histology grade had poorly or undifferentiated histology. The median cancer specific survival was 6 months (95% CI 4–9). The 1-year cancer-specific survival rate for entire cohort was 30.2%. There were no differences in terms of age at diagnosis, histological grade, tumor stage on presentation, and frequency of nephrectomy between carcinosarcoma of kidney (CSK) or renal pelvis (CSP). In multivariate analysis, age at diagnosis, tumor stage, and year of diagnosis were found to be significant predictors for cancer-specific survival. *Conclusion*. CSKP commonly presented as high-grade, advanced stage disease, and was associated with a poor prognosis regardless of location.

## 1. Background

Carcinosarcoma (CS) is a highly aggressive tumor composed of mixed malignant epithelial and mesenchymal components [1]. Carcinosarcoma of the kidney or renal pelvis (CSKP) is a rare malignancy of the genitourinary system. To date, fewer than 20 well-documented cases have been reported in the medical literature [2–10]. Most of the reported cases were carcinosarcoma of the renal pelvis (CSP) [2, 4–9]; only few cases were from kidney (CSK) [3, 10].

The histogenesis of carcinosarcomas remains a matter of controversy [3, 5, 8, 11]. Two main mechanisms were suggested: the “collision” or “multiclonal” hypothesis posits that the epithelial and mesenchymal components are distinct coexisting populations with different cells of origin [3]. The alternative “monoclonal” hypothesis suggests that carcinosarcoma arises from a single multipotent stem cell that differentiates along epithelial and mesenchymal pathways [11].

Due to the rareness of the disease, current literature on carcinosarcoma of the genitourinary system predominantly consists of a single case report and histopathological studies [2–10], the demographic features and clinical behavior of these tumors remain ill-defined; in addition, the survival of CSK and CSP was never directly compared.

In this study, a comprehensive analysis was performed to examine the epidemiology, natural history, and prognostic factors of patients with CSKP, and to determine if there was any difference between CSK and CSP in term of clinical presentation and outcome.

## 2. Methods

**Data Source**

The national Surveillance, Epidemiology, and End Results (SEER) database currently consists of 18 statewide and regional tumor registries spread throughout the US, covering approximately 26% of the population [12]. In this study, we used the SEER data based on the November 2007 submission. Data for this study was obtained from SEER Stat public-use data files, available from National Cancer Institute.

**Study Population**

The cases of carcinosarcoma were extracted from SEER on the basis of anatomic site (kidney 64.9, renal pelvis 65.9) and histologic type (ICD-Ocode 8980 and 8981). Patients over the age of 18 years, first diagnosed and/or treated between January 1973 and December 2007, were enrolled in this study.

### 2.1. Statistical Analysis

Patients were divided into 2 groups according to location (kidney versus renal pelvis). Student's *t*-test and the *χ* ^2^ test were, respectively, used for comparison of means and proportions between two groups. Survival duration was measured by the Kaplan-Meier method and compared by the log rank test [13]. Cases identified at the time of autopsy or by death certificate only were excluded from the survival analyses. Multivariable Cox proportional hazards model was used to identify independent predictors of long-term cancer specific death [14]. All statistical calculations were performed by SPSS 12.0 (Apache Software Foundation 2000). Comparative differences were considered statistically significant when the *P* value was <0.05.

## 3. Results

### 3.1. Patient and Tumor Characteristics

Forty-three patients with CSKP were identified in the SEER database during the study period, with a median age of 72 years (range 40–97). The majority of patients (88.4%) were white. Twenty-three percent of patients in the study were classified with distant stage disease. Except one patient, all the rest of patients with known histology had high-grade disease (poorly or undifferentiated histology). Overall, 74.4% of study subjects had radical nephrectomy, and 4.7% of patients had adjuvant radiation therapy.

Details regarding demographics, tumor characteristics, and treatment information are summarized in Table 1.

**Survival Analysis**

**Figure 1:**
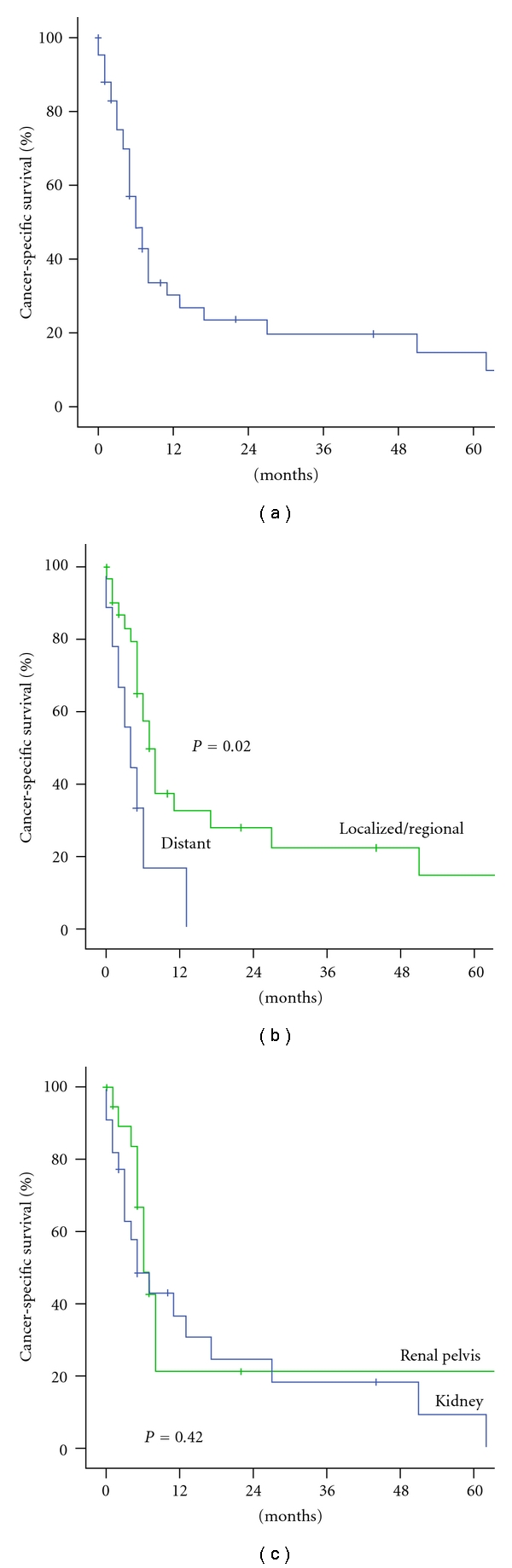
(a) Cancer-specific survival rate of patients with carcinosarcoma of the kidney and renal pelvis (CSKP). (b) Cancer-specific survival rate according to stage. (c) Cancer-specific survival rate according to tumor location.

For survival analyses, one patient that was diagnosed at the time of autopsy was excluded. A total of 42 patents were included in final survival analysis. The median duration of followup of the entire cohort was 5 months (range 0–146); 40 of 42 (95.2%) patients died during the follow-up period. The median cancer-specific survival was 6 months (95% CI 4–9; Figure 1(a)). The 1-year cancer specific survival rate for entire cohort was 30.2%. There was a significant difference of cancer-specific survival between patients with localized/regional disease and distant disease (*P* = 0.02; Figure 1(b)).

The median survival for CSK was 5 months (95% CI 1–9) and for CSP was 6 months (95% CI 4–8; Table 2). There were no significant differences in cancer-specific survival rate between CSP and CSK (*P* = 0.42; Figure 1(c)). In a multivariate survival analyses by Cox proportional hazard modeling, only age at diagnosis, tumor stage and year of diagnosis were identified as independent factors associated with cancer specific survival (Table 3).

## 4. Discussion

Most patients in this study presented with high histological grade and advanced-stage disease at the time of presentation: 79% of all patients with known SEER stage were classified as having regional or distant stage; almost all the patients (22 out of 23) with known histology grade had poorly or undifferentiated histology. The clinical and pathological characteristics of our study subjects were consistent with previous findings that CSKP is a very aggressive tumor [2–10]. These findings underscore the importance of early detection and diagnosis in this disease. 

Since there is no clinical trial specifically designed for CSKP, the optimal treatment strategy has not been established. Nephrectomy, radiation therapy, or chemotherapy has been used alone or in combination [3, 5]. Chen et al. reported the longest survival period, which is two years [8]. There was no survival benefit with the addition of postoperative chemotherapy, radiation, or combination [2, 5]. Consistent with single institution studies, the survival of patients in our study cohort was extremely poor. We did, however, observe a significant improvement in the outcome of patients with CSKP over time (Table 3). The improved outcomes over time likely reflect the advance in surgical techniques, and the increased adoption of multidisciplinary care. Further study to understand the molecular mechanism underlining the aggressive biological behavior of CSKP and the development of new strategies and aggressive therapeutic approaches for this tumor are urgently needed. 

Strengths of this study include the populations-based design, with cases from a broad spectrum of hospitals. Population studies are of particular importance for analysis of rare subtype cancer such as CSKP where, to date, no single institution study has enough cases to make meaningful analysis regarding important prognostic factors. 

Our findings have several limitations. First, the pathological diagnoses in SEER are based on local pathologists' reports and there is no central review of pathology reports. Unlike single-institution studies, the accuracy of staging and pathologic diagnosis within a national registry may vary widely across the institutions. Second, SEER data did not allow us to examine receipt of chemotherapy and patients' comorbidities, as well as performance status, all of which may influence survival in cancer patients. The analysis reported here attempted to overcome this data limitation by measuring cancer-specific survival, rather than overall survival. Finally, the sample size in our study may still not be large enough to fully describe the factors that affect the incidence, treatment choice, and survival of this rare malignancy.

## 5. Conclusions

In summary, CSKP is a highly malignant neoplasm, usually presented in elder males with an advanced stage at presentation and was rapidly lethal. A better understanding of the natural history of this disease and prognostic factors as provided herein are necessary to allow physicians and patients to accurately assess the risks and potential benefits of available therapy.

## Figures and Tables

**Table 1:** Demographic and clinical characteristics of 43 patients with carcinosarcoma of kidney and renal pelvis (CSKP).

Characteristics	Group	*N* (%)	Kidney (*n* = 23)	Renal Pelvis (*n* = 20)	*P*
Age mean (SD)			70 ± 11	71 ± 13	
Age	<75	27 (62.8%)	14	13	0.78
	≥75	16 (37.2%)	9	7	
Gender	Male	19 (44.2%)	11	8	0.16
	Female	24 (55.8%)	12	12	
Ethnicity	White	38 (88.4%)	19	19	0.22
	Nonwhite	5 (11.6%)	4	1	
Married	No	22 (51.2%)	13	9	0.45
	Yes	21 (48.8%)	10	11	
Grade	Low grade	1 (2.3%)	0	1	0.02
	High grade	22 (51.2%)	8	14	
	Unknown	20 (46.5%)	15	5	
SEER stage	Localized	7 (16.3%)	3	4	0.02
	Regional	24 (55.8%)	9	15	
	Distant	10 (23.3%)	9	1	
	Unstaged	2 (4.7%)	2	0	
Laterality	Left	23 (53.5%)	15	8	0.10
	Right	20 (46.5%)	8	12	
Nephrectomy	Yes	32 (74.4%)	14	18	0.05
	Unknown surgery	5 (11.6%)	5	0	
	No	6 (14%)	4	2	
Adjuvant Radiation	Yes	2 (4.7%)	2	0	0.28
	No	41 (95.3%)	21	20	
Year of diagnosis	1973–1988	7 (16.3%)	6	1	0.07
	1989–2004	36 (83.7%)	17	19	

High grade: poorly differentiated or undifferentiated.

**Table 2:** Median, 1-, 3-year cancer specific survival of patients with carcinosarcoma of kidney and renal pelvis (CSKP) according to demographic and clinical characteristics.

Characteristics	Median cancer specific survival months (95% CI)	Survival (%) 6 months, 1 year, 3 year			*P* value
All	6 (4–9)	48.4	30.2	19.6	
SEER stage					
Local/regional	7 (5–9)	86.6	79.4	22.4	0.02
Distant	4 (2–6)	16.7	16.7	0.00	
Nephrectomy					
No	3 (0–6)	40.0	20.0	20.0	0.77
Yes	7 (5–9)	50.9	31.3	18.8	
Location					
Kidney	5 (1–9)	48.3	36.8	18.4	0.42
Renal Pelvis	6 (4–8)	48.6	21.3	21.3	

**Table 3:** Multivariate analyses of factors associated with mortality in patients with carcinosarcoma of kidney and renal pelvis (CSKP).

Characteristics	Group	HR	(95% CI)	*P* value
Age	<75	1.00	(1.19–8.33)	0.02
	≥75	3.16		
Gender	Female	1.00	(0.90–4.82)	0.085
	Male	2.08		
SEER stage	Distant	1.00	(.096–0.74)	0.011
	Localized/regional	0.27		
Location	Renal pelvic	1.00	(0.29–1.63)	0.40
	Kidney	0.69		
CDS	No	1.00	(0.29–5.64)	0.75
	Nephrectomy	1.28		
Diagnosis year	1973–1984	1.00	(0.02–0.77)	0.025
	1985–2004	0.13		

HR: harzard ratio; SEER: Surveillance, Epidemiology, and End Results.

CDS: cancer directed surgery.
